# Modulation of Notch signaling pathway in activated hepatic stellate cells does not ameliorate the outcome of liver fibrosis in carbon tetrachloride and DDC-feeding models

**DOI:** 10.3389/fphar.2024.1440236

**Published:** 2024-10-28

**Authors:** Dino Šisl, Pavao Planinić, Sanja Novak, Maša Filipović, Darja Flegar, Alan Šućur, Petra Turčić, Nataša Kovačić, Ivo Kalajzić, Danka Grčević, Tomislav Kelava

**Affiliations:** ^1^ Laboratory for Molecular Immunology, School of Medicine, Croatian Institute for Brain Research, University of Zagreb, Zagreb, Croatia; ^2^ Department of Physiology, School of Medicine, University of Zagreb, Zagreb, Croatia; ^3^ Department of Physiology, School of Medicine, University of Mostar, Mostar, Bosnia and Herzegovina; ^4^ Department of Reconstructive Sciences, University of Connecticut Health Center, Farmington, CT, United States; ^5^ Department of Pharmacology, Faculty of Pharmacy and Biochemistry, University of Zagreb, Zagreb, Croatia; ^6^ Department of Anatomy, School of Medicine, University of Zagreb, Zagreb, Croatia

**Keywords:** Notch, liver fibrosis, CCl4, DDC, transgenic mice

## Abstract

**Background:**

Recent research suggests a possible role of Notch signaling pathway in development of liver fibrosis, but exact cellular and molecular mechanisms are still not well defined. Methods: We modulated Notch signaling in activated hepatic stellate cells/myofibroblasts using the model of inducible activation or inhibition of Notch signaling selective for αSMA positive cells in murine models of toxic fibrosis induced by CCl4 and cholestatic fibrosis induced by DDC supplemented feeding.

**Results:**

Our results confirm that Notch signaling pathway is activated in both CCL4 and DDC model of liver fibrosis and that αSMA positive myofibroblasts are of activated hepatic stellate cells origin. However, neither the inhibition of canonical Notch signaling (in tamoxifen treated αSMACreER/RBP-J^fl/fl^ mice) nor its overactivation (in tamoxifen treated αSMACreER/NICD1 mice) changed the degree of liver fibrosis in comparison to the control groups in either of the investigated models. Furthermore, after the withdrawal of the fibrogenic treatment the degree of resolution of fibrosis was similar between the animals with Notch overactivation and controls. In addition to genetic manipulation, we investigated the effect of antibodies against NOTCH1 and NOTCH2 on the development of liver fibrosis. Treatment with antibodies had effects on thymus and spleen respectively, but failed to ameliorate liver fibrosis. In conclusion, our data demonstrate that modulation of Notch activity in activated HSC is not sufficient to change the outcome of liver fibrosis. The results obtained with inhibitory antibodies further demonstrate limitations of targeting Notch 1 and 2 receptors as antifibrotic therapy. Notch pathway remains a potential target for the treatment of liver fibrosis, but future studies should be directed to Notch 3 signaling and/or targeting different populations of cells.

## Introduction

Liver fibrosis is a common feature of various chronic liver diseases. During persistent hepatic injury, quiescent hepatic stellate cells (HSC) are activated and differentiate into alpha smooth muscle actin (αSMA)-positive myofibroblasts that deposit collagen into the extracellular matrix. Initially the process is reversible, but under sustained injury, disease will progress toward irreversible cirrhosis (Troeger et al., 2012; Mederacke et al., 2013; Kim et al., 2024). Various signaling pathways play a role in the activation and transformation of HSCs into the collagen producing myofibroblasts with a predominant role of transforming growth factor beta (TGFβ) and platelet-derived growth factor (PDGF) (Yoshida and Matsuzaki, 2012; Greuter and Shah, 2016). The findings of the recently published research suggest a possible participation of Notch signaling pathway in HSC activation (Valizadeh et al., 2021).

Notch signaling pathway is an evolutionary conserved system that consists of five ligands and four receptors. Upon activation of the canonical pathway, Notch receptors undergo cleavage of the Notch intracellular domain (NICD), enter the nucleus where they bind recombination signal binding protein immunoglobulin kappa J (RBPJ), resulting in activation of transcription of numerous target genes including *Hes* and *Hey* (Jarriault et al., 1995; Sucur et al., 2020). The essential role of Notch during the embryonic development of the liver has been well established and changes in Notch signaling are observed in various types of chronic liver injuries leading to liver fibrosis (Kodama et al., 2004; Adams and Jafar-Nejad, 2019). For example, Tanaka et al. reported a higher expression of Notch 2 in patients with primary biliary cirrhosis (Tanaka et al., 2001) and upregulation of Notch signaling pathway was reported in various human chronic liver diseases and animal models of liver injuries (Adams and Jafar-Nejad, 2019).

The results of the study conducted by Chen et al. indicated that activation of Notch signaling might prevent the development of liver fibrosis by inhibiting activation of HSC whereas the blockade of Notch signaling with Notch1 siRNA was associated with a greater proliferation of cultured stellate cells (Chen et al., 2011). On the other hand, findings of an *in vivo* study with pharmacological inhibition of Notch pathway by application of the gamma secretase inhibitors indicated that Notch inhibition has an antifibrotic effect in carbon tetrachloride (CCl4) model of liver fibrosis (Chen et al., 2012). Xie et al. (2013) showed similar effect of gamma secretase inhibitors on aHSC *in vitro* Lee et al. (2015) found antifibrotic effect of synthetic decoy oligodeoxynucleotide for RBP-J in a DDC model of liver fibrosis. Furthermore, similar findings were reported for inhibition of Notch ligand Jagged1 in a CCl4 model (Tang et al., 2017).

The effects of Notch signaling alterations can be specific to a cell lineage. As nonselective inhibition of Notch is associated with multiple side effects (Previs et al., 2015), it is essential to precisely identify cell populations in which Notch modulation may be applied to alleviate hepatic fibrogenesis. Findings of various studies, mainly conducted on models of fatty liver disease indicate that antifibrotic effect may be achieved by selective modulation of Notch signaling in various cells types, including hepatocytes, liver sinusoidal endothelial cells (LSEC), myeloid cells, and macrophages (Duan et al., 2018; Zhu et al., 2018; Ding et al., 2023 Song et al., 2023).

So far, only one report on Notch signaling pathway regulation in activated HSC/myofibroblasts *in vivo* was conducted by Yue et al. in the model of liver fibrosis induced by CCl4 in which the authors used SMA22αCreER mice to inhibit Notch signaling after tamoxifen treatment and reported reduced degree of fibrosis (Yue et al., 2021).

Alpha smooth muscle actin (*Acta2*, αSMA) is expressed in HSC upon injury at a typical sites of fibrosis development. We decided to use a comprehensive approach to modulate Notch signaling in myofibroblasts using the model of inducible activation or inhibition of Notch signaling in murine models of toxic fibrosis induced by CCl4 and cholestatic fibrosis induced by DDC supplemented feeding.

## Methods

### Animals

All animal experiments in this study were conducted under protocols approved by the Croatian national Ethics Committee (EP 168/2018). Mice were maintained at the animal facility of the Croatian Institute for Brain Research, University of Zagreb School of Medicine (Zagreb, Croatia) under standard housing conditions. All experiments were performed on male 8–12 weeks old mice. We used wild type C57Bl/6 mice to establish CCl4 and DDC-supplementation models of fibrosis. For visualization of αSMA positive cells we used previously developed αSMACreER mice (Grcevic et al., 2012) and crossed them with Ai9 reporter mice (B6.Cg-Gt (ROSA) 26Sortm9(CAG-tdTomato) Hze/J) that express tdTomato fluorescent protein after Cre-mediated activation following the tamoxifen injection. To study the effects of Notch inhibition in αSMA + cells we used αSMA CreER mice and bred them with RBP-J floxed mice (Rbpjtm1Hon, provided by professor Tasuku Honjo, Kyoto University). The offspring were bred again with RBP-J floxed mice to obtain Cre+ and Cre− littermates, homozygous for the floxed RBP-J allele (αSMACreER/RBP-J^fl/fl^ mice). To generate mice overexpressing Notch1 signaling in the αSMA + cells we used αSMA CreER mice and bred them with NICD1 floxed mice (Gt (ROSA) 26Sortm1(Notch1) Dam/J from the Jackson Laboratory, stock no. 008159) to produce αSMACreER/NICD1. In all experiments we used Cre+ and Cre-littermates treated with tamoxifen, with Cre-being a control to identically treated Cre + animals.

### Carbon-tetrachloride model

Mice were injected two times per week by CCl4 (cat. No. 289116, Sigma-Aldrich) diluted 1:3 in corn oil (cat. No. C8267, Sigma-Aldrich) at a dose of 1 mL/kg (ip) and sacrificed after 2–6 weeks as indicated in the result section. In experiments that involved αSMACreER/RBP-J^fl/fl^ and αSMACreER/NICD1 mice Cre+ and Cre-littermates received tamoxifen (cat. No. T5648, Sigma-Aldrich, 75 μg/g in corn oil, ip, 2x per week) 1 day after CCl4 injection. In experiments where the effect of Notch overactivation on resolution of liver fibrosis was studied Cre+ and Cre- αSMACreER/NICD1 littermates were treated for 3 weeks in the same manner (CCl4 and Tamoxifen given 2x per week) and then allowed to recover for 4 weeks until sacrifice.

### DDC model of cholestatic fibrosis

Standard food pellets (4RF21) and same pellets supplemented with 0.025% DDC (Sigma-Aldrich, Burlington, MA, United States, cat. No.: 137030) were obtained from Mucedola (Milan, Italy). Mice were fed with standard pellets or DDC-supplemented pellets and sacrificed after 2–4 weeks as indicated in the results. In the experiments that involved αSMACreER/RBP-J^fl/fl^ and CreER/NICD1 mice Cre+ and Cre-littermates received Tamoxifen 3x per week (75 μg/g in corn oil, ip). In experiments where the effect of Notch overactivation on resolution of liver fibrosis was studied Cre+ and Cre- αSMACreER/NICD1 littermates were treated for 2 weeks and then allowed to recover for 4 weeks until sacrifice.

### Application of anti-Notch antibodies

Antibodies against NOTCH1 (anti-mouse NRR1), and NOTCH2 (anti-mouse NRR2) receptors as well as a control antibody (Ragweed; 9652 10D9.W. stable mIgG2a) (Wu et al., 2010) were obtained from Genentech (San Francisco, CA, United States) in accordance with material transfer agreement (ID: OR-224641). In the initial experiment all antibodies were applied at a dose of 2 mg/kg, ip 2x per week). The dose of anti-Notch1 was reduced to 1 mg/kg due to negative effects on health of the fibrotic animals (described in the results).

### Isolation and purification of hepatic stellate cells

For isolation of HSCs we used a protocol described by Mederacke et al. (2015). Briefly, mice were anesthetized, and livers were perfused through inferior vena cava with the following solutions: EGTA solution (1–2 min) pronase solution (cat. No. P5147, Sigma-Aldrich) for 7 min and collagenase solution (cat. No. 11088882001, Roche, Basel, Switzerland) for 5 min at 5 mL/min. Digested livers were further incubated for 30 min in collagenase/pronase solution and centrifuged on a Nycodenz (cat. No. AN 1002423, Accurate Chemicals, Westbury, NY, United States) gradient. The purity of isolated cells was confirmed by fluorescent microscopy by confirming the transient fluorescence of isolated cells under UV light. Further purification of isolated HSC was performed using the retinoid based FACS sorting (BD FACSAria II (BD Biosciences instrument) using the violet 405-nm laser for excitation and a 450/50-nm band-pass filter for detection (Mederacke et al., 2015). In some experiments isolated HSCs were cultured in DMEM medium (Gibco, Cat No. 21063029) supplemented with 10% fetal bovine serum, 2 mM L-glutamine, and 1% penicillin/streptomycin. Wells were further supplemented with 1 µM hydroxytamoxifen (4-OHT, cat. No. 68047-06–3, Sigma) or vehicle (ethanol).

### Histology—Sirius red staining

Samples of liver tissue were fixed in 4% paraformaldehyde overnight and dehydrated in increasing ethanol concentrations (70%, 96%, and 100%). Following transfer to benzene for 30 min, samples were embedded in paraffin overnight. Sections were cut (5 µm) with a rotational microtome (Leica SM 2000 R, Leica Biosystems, Nussloch, Germany), and finally stained with Sirius red dye. Slides were analyzed and photographed under a light microscope (Axiovert 200; Carl Zeiss, Oberkochen, Germany) equipped with a camera. Photographs were taken, and the red-stained surface area was quantified using the ImageJ (NIH) processing program.

### Determination of serum activity of aminotransferases

The serum was separated by centrifugation after clot formation and stored at −20°C until analysis. The alanine-aminotransferase (ALT) and aspartate-aminotransferase (AST) serum levels were determined by standard laboratory techniques in a clinical diagnostic laboratory using an Olympus AU400 analyzer as described previously (Markotic et al., 2020).

### Determination of hydroxyproline content in liver tissue

The hydroxyproline content in the liver tissue was determined using a commercial kit according to the manufacturer’s instruction (Hydroxyproline Assay Kit, cat. No. MAK008, Sigma-Aldrich) with slight modification as described previously (Šisl et al., 2022). Briefly, livers were homogenized in ultrapure water (10 mg of tissue per 100 µL of water) and hydrolyzed in 12 M HCl (at 100°C for 20 h). After centrifugation (100,00× g for 3 min, room temperature) the supernatant was transferred to a new tube and dried under a vacuum at 60°C. After incubation in 100 µL of Chloramine T/Oxidation Buffer Mixture at room temperature for 5 min, 100 µL of freshly diluted 4-(dimethylamino) benzaldehyde (DMAB) reagent was added. After incubation (90 min at 60°C), the absorbance was read at 560 nm, and the concentration was determined using a standard curve obtained by the measurement of hydroxyproline standards provided in the kit.

### Flow cytometry

Single cell suspensions of spleen and thymus were prepared and stained with the antibody panels to identify marginal zone B lymphocytes (anti-mouse CD23, anti-mouse CD21/CD35, anti-mouse CD19) or double-positive thymocytes (anti-mouse CD4 and anti-mouse CD8) using directly conjugated monoclonal antibodies. Upon labelling, cells were acquired and analyzed using the BD FACSAria II (BD Biosciences) instrument and FlowJo software (FlowJo).

### Quantitative PCR gene expression analysis

Total RNA was isolated from the liver tissue samples using TRI reagent (cat. no. T9424, Sigma-Aldrich) and quantified on a Nanodrop spectrophotometer (Thermo Fisher Scientific, Waltham, MA, United States). A High-Capacity RNA-to-cDNA Kit (Applied Biosystems) was used for reverse transcription and generation of cDNA. The cDNA was amplified by ABI Prism 7500 system (Applied Biosystems, Waltham, MA, United States) using TaqMan Gene Expression Master Mix (cat. no. 4369514, Applied Biosystems, Waltham, MA, United States) and commercially available TaqManTM Gene Expression Assays (Applied Biosystems, Waltham, MA, United States) for *Col1a1* (Assay ID: Mm00801666_g1), *Acta2* (Assay ID: Mm01546133_m1), *Hes1* (Assay ID: Mm01342805_m1), *Hey1* (Assay ID: Mm00468865_m1), *HeyL* (Assay ID: Mm00516555_m1), *Notch1* (Assay ID: Mm00627185_m1), *Notch2* (Assay ID: Mm00803077_m1), *Notch3* (Assay ID: Mm01345646_m1), *Jag1* (Assay ID: Mm00496902_m1), *Jag2* (Assay ID: Mm01325629_m1), *Dll4* (Assay ID: Mm00444619_m1), keratin 19 (*Krt19*. Mm00492980_m1), lecithin-retinol acyltransferase (*Lrat,* Mm00469972_m1) and *Gapdh* (AssAy ID: Mm99999915_g1). SYBR green real-time qPCR in conjunction with primers for RBPJ exon 1-2 and exon 6-7 was utilized as described (Delacher et al., 2019). Gene expression was calculated using the ∆∆CT method and normalized to the expression level of the housekeeping gene and normalized to control group. To confirm recombination in tamoxifen treated αSMACreERT2/NICD1 mice DNA was isolated from hepatic stellate cells and recombined fragment of *Nicd1* was confirmed by PCR as described previously (Novak et al., 2020).

### Statistical analysis

Data are presented as mean ± standard deviation and analyzed using Student's t test/ANOVA or Mann-Whitney test, as appropriate. Post hoc tests and multiple comparisons were corrected by Dunnett. All tests were two-tailed, and P < .05 was considered statistically significant. GraphPad Prism version 6 for Windows (GraphPad Software Inc.) software was used for analysis.

## Results

### Notch signaling is modulated in CCl4 and DDC-models

**FIGURE 1:**
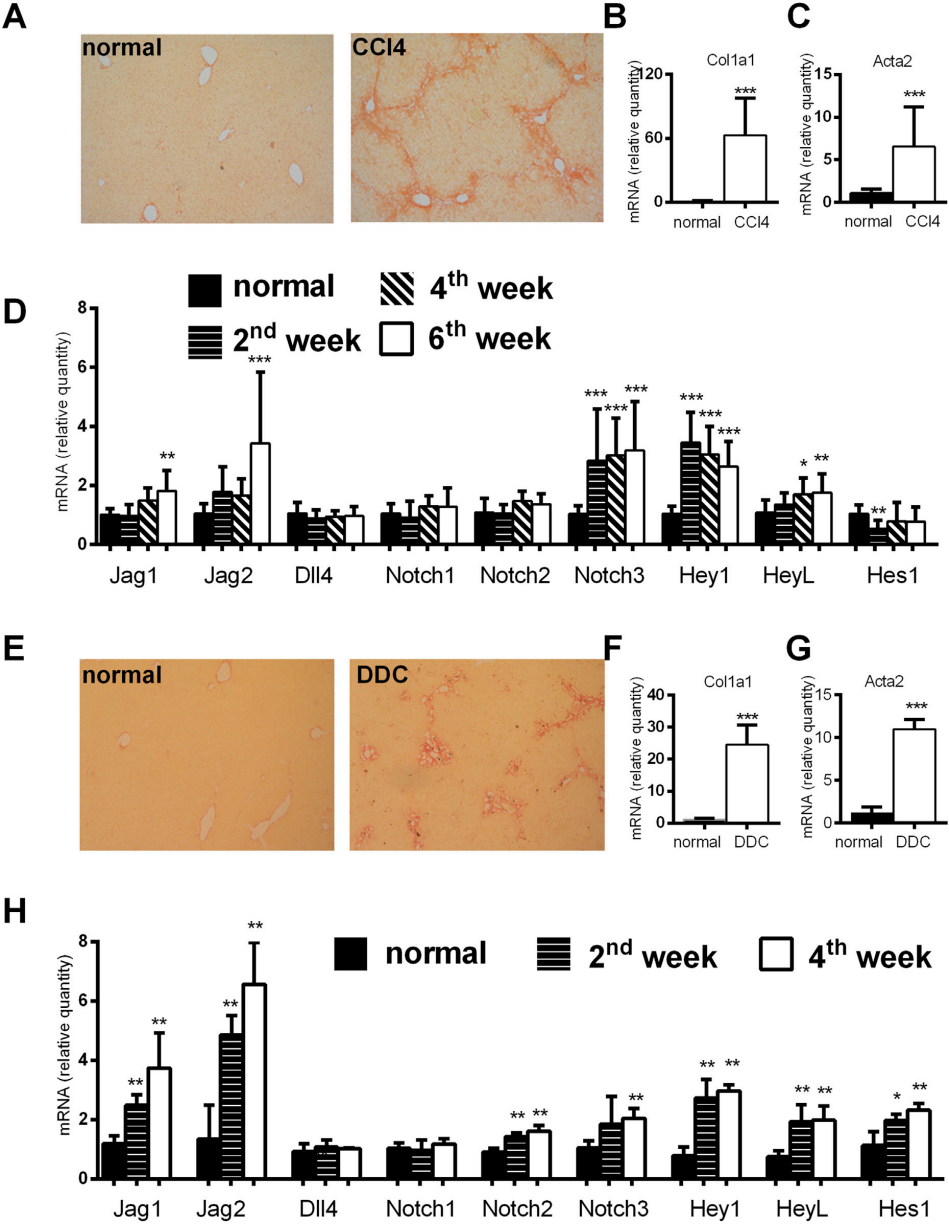
Analysis of histological and genetic changes in a CCl4 and DDC model of liver fibrosis. **(A–D)** Mice were injected with CCl4 (2x per week) and sacrificed at indicated time points. Six weeks of CCl4 treatment caused an increase in Sirius red stained area **(A)**; and increased expression of *Col1a1* **(B)**; and *Acta2* **(C)**; expression of Notch related genes was upregulated at various time points **(D)**. **(E–H)** Mice were fed normal or DDC-supplemented diet and sacrificed at indicated time points. Four weeks of DDC treatment caused an increase in Sirius red stained area **(E)**; increased expression of *Col1a1* **(F)**; and *Acta2* **(G)**; expression of Notch related genes was upregulated at various time points **(H)**. Data represent mean +standard deviation, (n = at least 6 mice per group per time point) ANOVA test with Dunnett’s *post hoc* correction was used for comparison between the groups, *p < 0.05, **p < 0.01, ***p < 0.001 compared to control group, NS—nonsignificant, CCl4—Carbon tetrachloride, DDC—3,5-diethoxycarbonyl-1,4-dihydrocollidine.

We established animal models of CCl4-and DDC-induced fibrosis and analyzed expression of Notch related genes. Both treatments caused liver fibrosis associated with an increased expression of *Col1a1* and *Acta2* in liver tissue. We determined expression of Notch signaling genes and its downstream targets. Analysis of Notch signaling related genes showed an increase in expression of *Jag1*, *Jag2*, *Notch3*, *Hey1*, *HeyL* in the CCl4 model whereas increased expression of *Jag1* and *Jag2* ligands, *Notch2* and *Notch3*, and *Hey*1, *HeyL*, *Hes1*, was observed in the DDC model (Figure 1).

**FIGURE 2:**
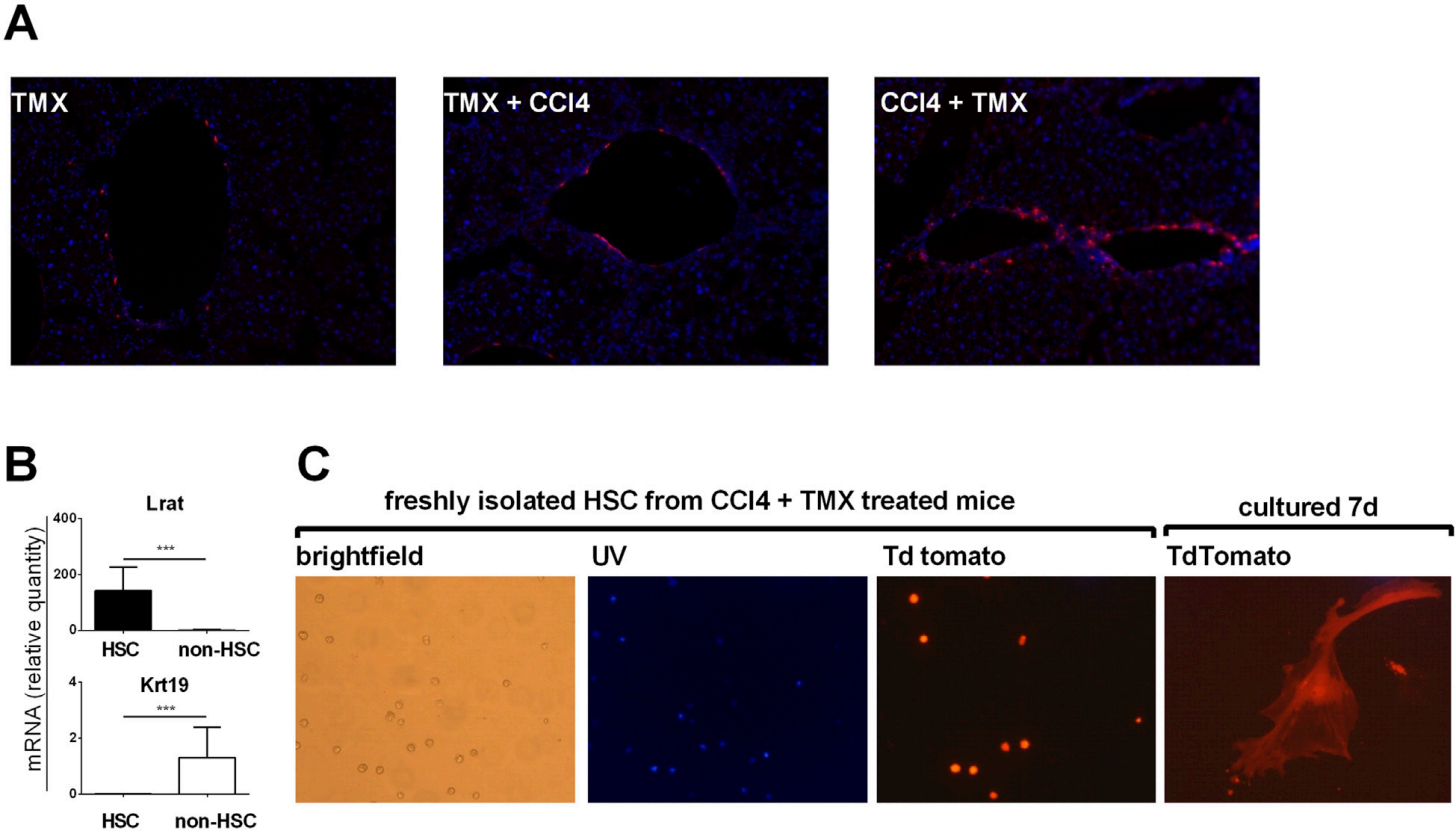
Myofibroblasts in liver fibrosis are of hepatic stellate cells origin. **(A)** Cryosections of liver tissue from aSMACreERT/Ai9 were stained with DAPI (blue). In αSMACreERT/Ai9 mice that received two injections of tamoxifen only vascular smooth muscle cells are tdTomato positive (**TMX**). Similar pattern is visible in mice that received two injections of tamoxifen 5 and 7 days before the beginning of the CCl4 treatment (2x/week for 3 weeks) (**TMX + CCl4**). In mice that received tamoxifen on the third and fifth day after the beginning of CCl4 treatment tdTomato positive cells were found on typical sites of fibrosis development (**CCl4 + TMX**). **(B)**, Expression of genes for *LRaT* and *Krt19* in retinoid positive hepatic stellate cells (HSC) and retinoid negative (non hepatic cells population) **(C)** Hepatic stellate cells isolated from CCl4 + TMX treated αSMACreERT/Ai9 were transiently autofluorescent (Vitamin A positive) under the UV light, with tdTomato positivity found in isolated cells. Cultured *in vitro* they differentiated into the tdTomato positive myofibroblasts after 7 days of culture.

To confirm that majority of αSMA positive myofibroblasts are of HSC origin we employed αSMACreER/Ai9 mice. In line with results of Mederacke et al. we showed that αSMA labeled only a few vascular smooth muscle cells within the liver when tamoxifen was applied to untreated mice. When tamoxifen was applied 1 week before fibrosis induction, similar pattern was visible suggesting that there was no proliferation and differentiation of cells initially positive for αSMA. However, when tamoxifen was applied after the induction of fibrosis (at the moment when HSC are activated and begin to express αSMA) TdTomato labeled cells were found on typical sites of fibrosis development (Figure 2A). Next, we established protocol for isolation of HSCs and performed gene expression analysis on sorted HSCs. Analysis confirmed increased expression of HSC-specific gene *Lrat* in retinoid positive population with a decrease in cholangiocyte-specific gene *Krt19* compared to retinoid-negative non-HSC population (Figure 2B). By isolation of HSC from fibrotic livers of αSMACreER/Ai9 mice we further confirmed that TdTomato labeled cells are activated HSC able to differentiate *in vitro* into myofibroblasts (Figure 2C).

### Notch inhibition in activated hepatic stellate cells does not change the degree of the fibrosis in CCL4 and DDC-models

**FIGURE 3:**
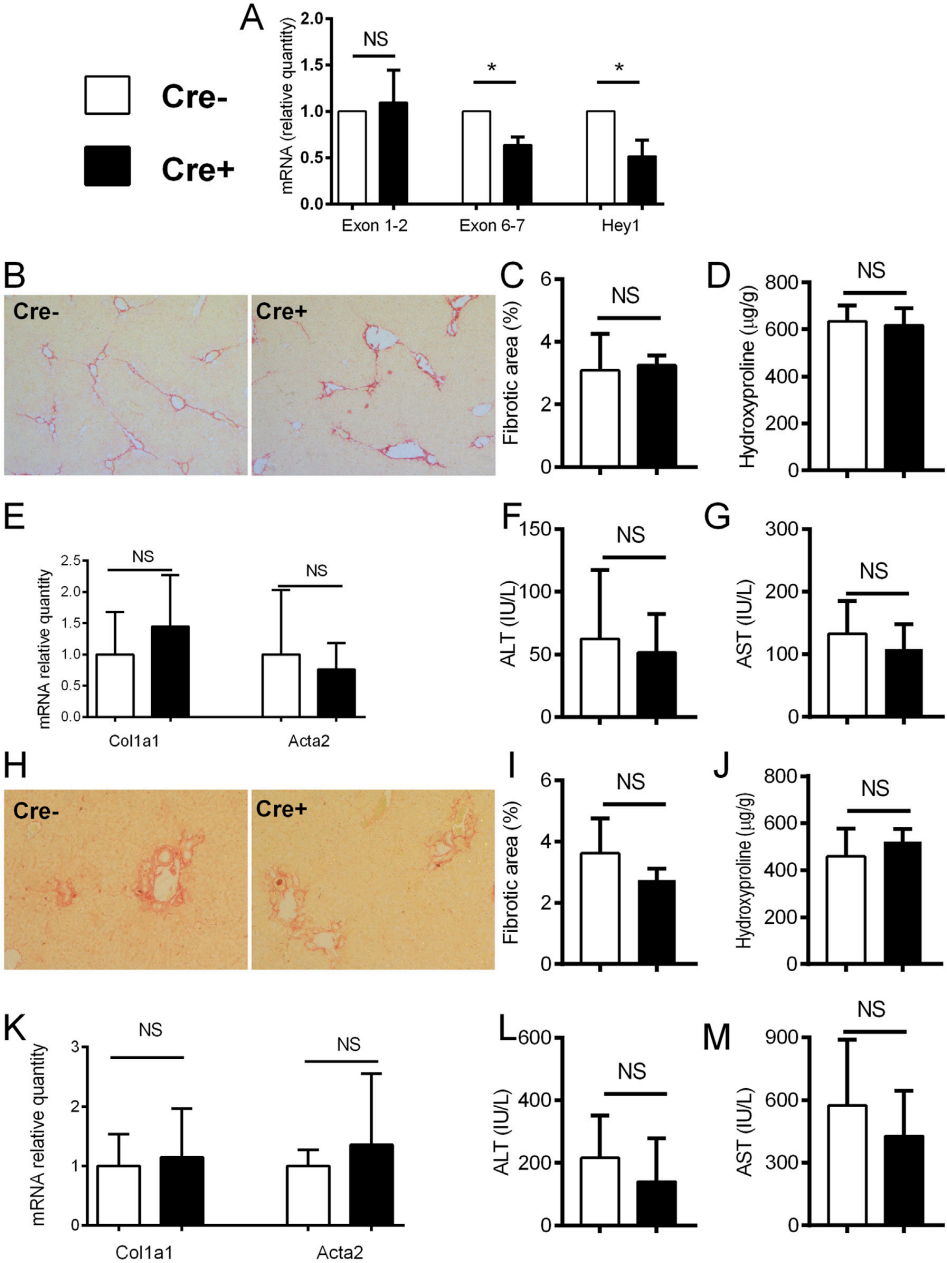
Notch inhibition in αSMA cells does not change the degree of fibrosis. **(A)** Cre^−^ and Cre^+^ αSMACreER/RBP-J^fl/fl^ mice were treated with CCl4 and TMX (2 x per week) for 2 weeks, HSC were isolated and gene expression was analyzed by PCR (n = 3 per group). **(B–G)**, Cre^−^ and Cre^+^ αSMACreER/RBP-J^fl/fl^ mice were treated with CCl4 and TMX (2 x per week) for 6 weeks, Sirius red area **(B, C)**, hydroxyproline content in liver **(D)**, expression of genes *Col1a1* and *Acta2* **(E)** and ALT **(F)** and AST **(G)** aminotransferase activity in plasma were analyzed (n = 12-15 per group). **(H–M)**, Cre^+^ and Cre^−^ αSMACreER/RBP-J^fl/fl^ mice were fed with DDC-diet and treated with TMX (3 x per week) for 4 weeks, sirius red area **(H, I)**, hydroxyproline content in liver **(J)**, expression of genes *Col1a1* and *Acta2* **(K)** and ALT (**L**) and AST (**M**) aminotransferase activity in plasma were analyzed (n = 8-10 per group). Data represent mean with standard deviation, Student T test was used for comparison between the groups. *p≤ 0.05, NS—non-significant.

After establishing increased Notch signaling during liver fibrosis, we aimed to further assess whether Notch inhibition in activated HSCs will affect the development of hepatic fibrosis. To disable Notch canonical signaling we used αSMACreER/RBP-J^fl/fl^ mice, and applied tamoxifen treatment to induce *Rbp-j* deletion after the beginning of the CCl4 treatment. We confirmed gene recombination as evidenced by decreased expression of floxed exon 6-7 of the *Rbp-j* gene and Notch regulated *Hey1* in Cre + animals upon CCl4/tamoxifen treatment. No difference in the expression of exon 1-2 which is located upstream of the floxed region was observed (Figure 3A). However, lack of Notch canonical signaling in Cre + animals did not affect the fibrosis level compared to Cre-animals as evidenced by similar size of the sirius red stained area in histological sections, content of hydroxyproline in the liver tissue, activity of aminotransferases in plasma, and expression of *Col1a1* and *Acta2* in liver tissue. (Figures 3B–G). Similarly as in the CCl4 model, we have not observed differences in the degree of liver fibrosis, hydroxyproline content, aminotransferase activities and *Col1a1/Acta2* between Cre+ and Cre-mice in the DDC model (Figures 3H–M).

### Forced activation of Notch in activated hepatic stellate cells does not change the degree of the fibrosis in CCL4 and DDC-models

**FIGURE 4:**
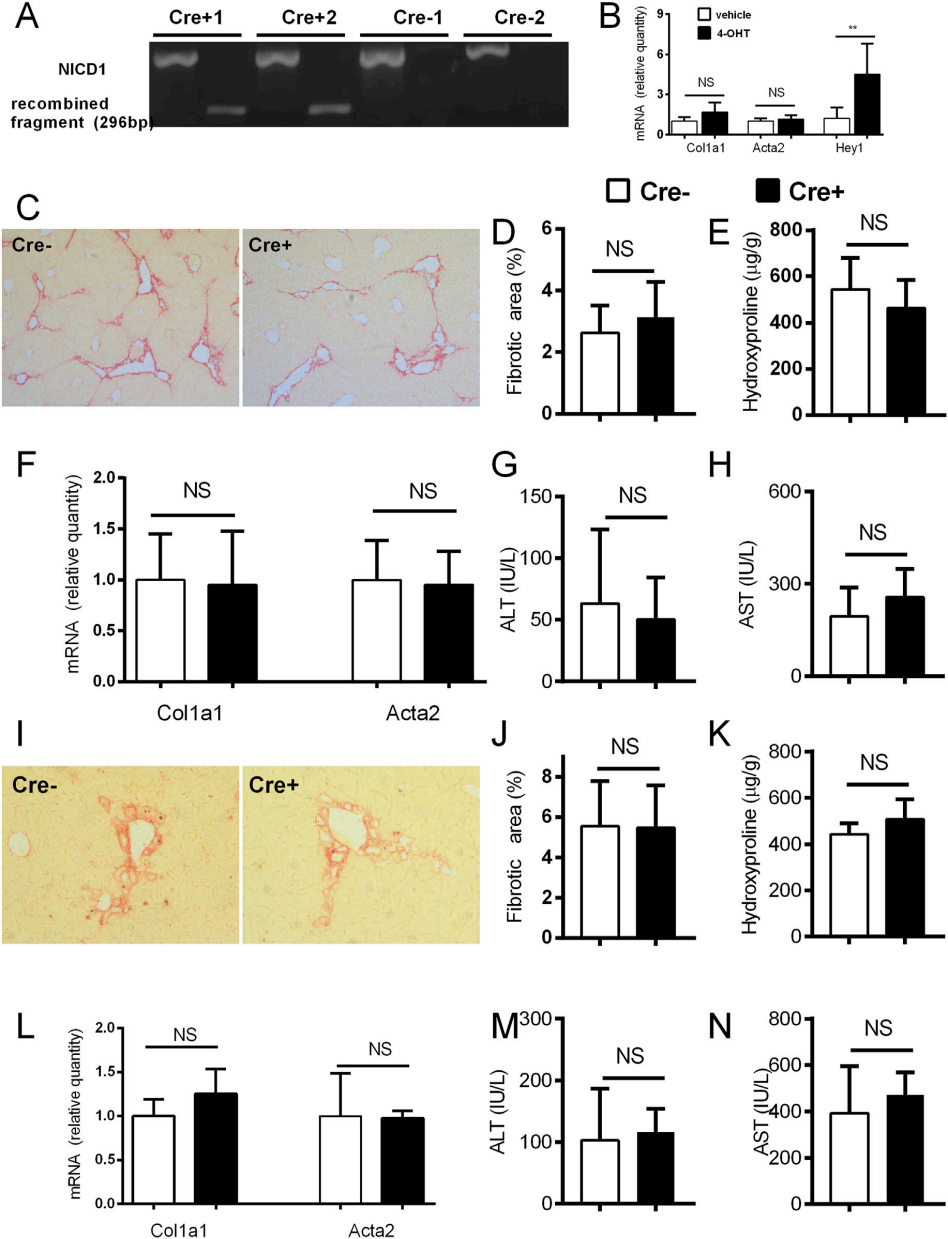
Forced activation of Notch1 in αSMA cells does not change the degree of fibrosis. **(A)** Cre^−^ and Cre^+^ αSMACreERT2/NICD mice were treated with CCl4 and TMX (2 x per week) for 2 weeks, HSC were isolated and DNA was analyzed by PCR. **(B)** Isolated HSC from Cre+ αSMACreERT2/NICD mice were cultured for 7 days with addition of 4-OHT or vehicle and expression of genes for *Col1a1*, *Acta2* and *Hey1* was analyzed (n = 4 per group). **(C–H)** Cre^−^ and Cre^+^ αSMACreERT2/NICD mice were treated with CCl4 and TMX (2 x per week) for 6 weeks: sirius red area **(C**,**D)**, hydroxyproline content in liver **(E)**, expression of genes *Col1a1* and *Acta2* **(F)** and ALT **(G)** and AST **(H)** aminotransferase activity in plasma were analyzed (n = 8-15 per group). **(I–N)**, Cre^−^ and Cre^+^ αSMACreERT2/NICD mice were fed with DDC-diet and treated with TMX (3 x per week) for 4 weeks, Sirius red area **(I, J)**, hydroxyproline content in liver **(K)**, expression of genes *Col1a1* and *Acta2* **(L)** and ALT **(M)** and AST **(N)** aminotransferase activity in plasma were analyzed (n = 6-8 per group). Data represent mean with standard deviation, Student T test was used for comparison between the groups. **p≤ 0.01, NS—non-significant.

In the experiments on αSMACreER/NICD1 mice, the recombination of DNA in the HSC of the Cre + animals upon CCl4/tamoxifen treatment was confirmed by PCR with no recombination in Cre-animals upon the identical treatment (Figure 4A). *In vitro* addition of 4-OHT to the cultured HSC from the Cre+ αSMACreER/NICD1 animals significantly increased expression of *Hey1* confirming the Notch overactivation, but had no influence on *Col1a1* and *Acta2* genes, associated with HSC activity (Figure 4B). In *in vivo* experiments, there was no difference in the histological degree of liver fibrosis between the Cre+ animals with overactiavated Notch1 signaling compared to Cre-controls (Figures 4C, D). This finding was confirmed by no difference in hydroxyproline content, aminotransferase activity and *Col1a1* and *Acta2* expression between the groups (Figures 4E–H). Similar results with no difference between the groups in none of the investigated fibrotic parameters were obtained in the DDC model (Figures 4I–N).

### Forced activation of Notch in activated hepatic stellate cells does not impair resolution of liver fibrosis

**FIGURE 5:**
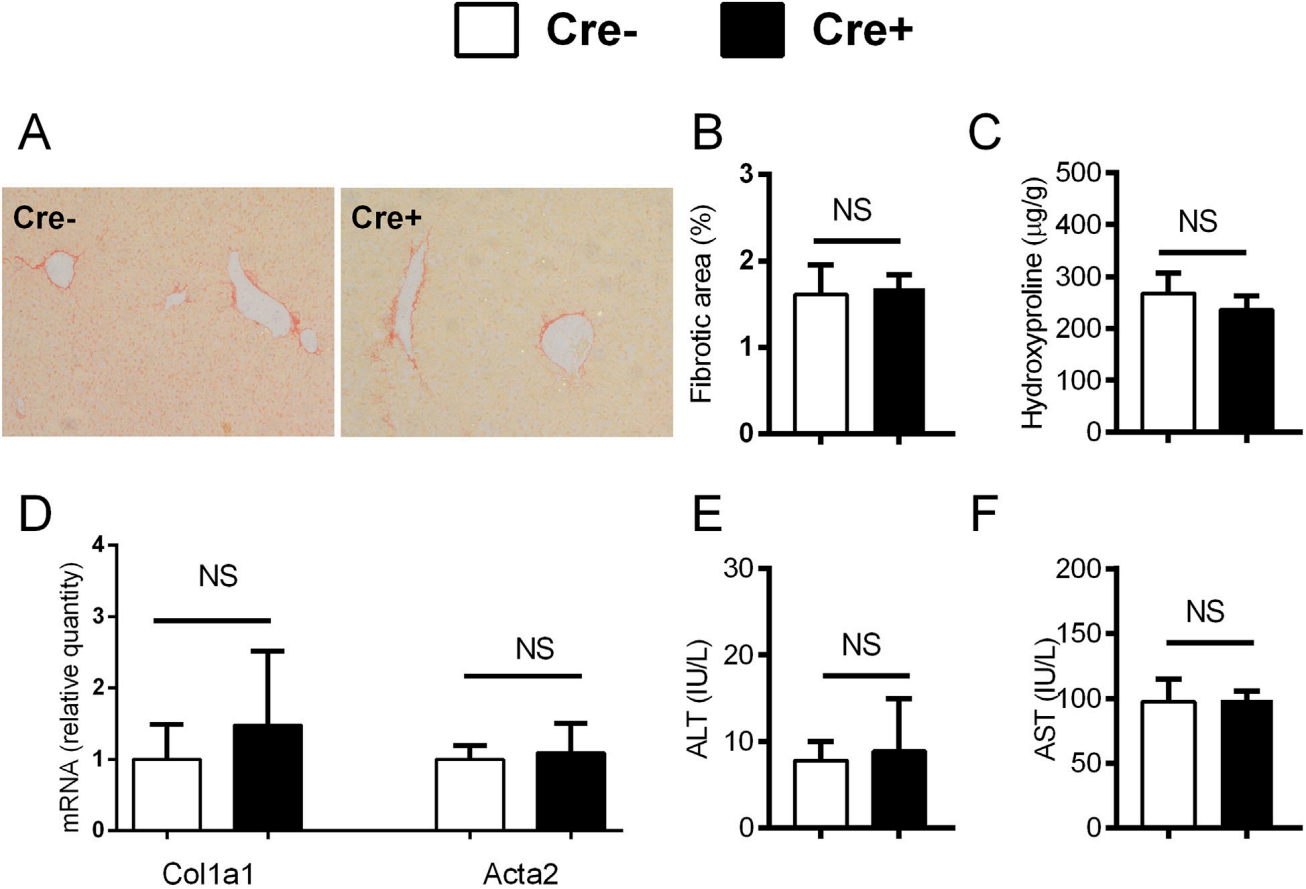
Forced activation of Notch1 in αSMA cells does not interfere with the recovery from fibrosis in CCl4 model. **(A–F)** Cre^−^ and Cre^+^ αSMACreERT2/NICD1 mice were treated with CCl4 and TMX (2 x per week) for 3 weeks and then allowed to recover for 4 weeks. Sirius red area **(A, B)**, hydroxyproline content in liver **(C)**, expression of genes *Col1a1* and *Acta2* **(D)** and ALT **(E)** and AST **(F)** aminotransferase activity in plasma were analyzed (n = 6-9 per group). Data represent mean with standard deviation, Student T test was used for comparison between the groups. NS—non-significant.

We investigated whether the forced Notch activation interferes with resolution of fibrosis after elimination of the profibrotic substance. Mice were treated with CCl4 for 3 weeks and then allowed a period of 4 weeks recovery. After the period of recovery measured parameters returned to levels comparable to healthy control although remnants of fibrosis were mildly discernable on histological findings. The degree of recovery was similar between the Cre+ and Cre- αSMACreER/NICD1 animals (Figures 5A–F). Similar results were obtained in the DDC model (see Supplementary Material, Supplementary Figures S2A–F) suggesting that overactivation of Notch in HSC and vascular smooth muscle cells did not interfere with resolution of fibrosis in either of the investigated models.

### Treatment with antibodies against Notch1 and Notch2 receptors does not ameliorate fibrogenesis in liver

**FIGURE 6:**
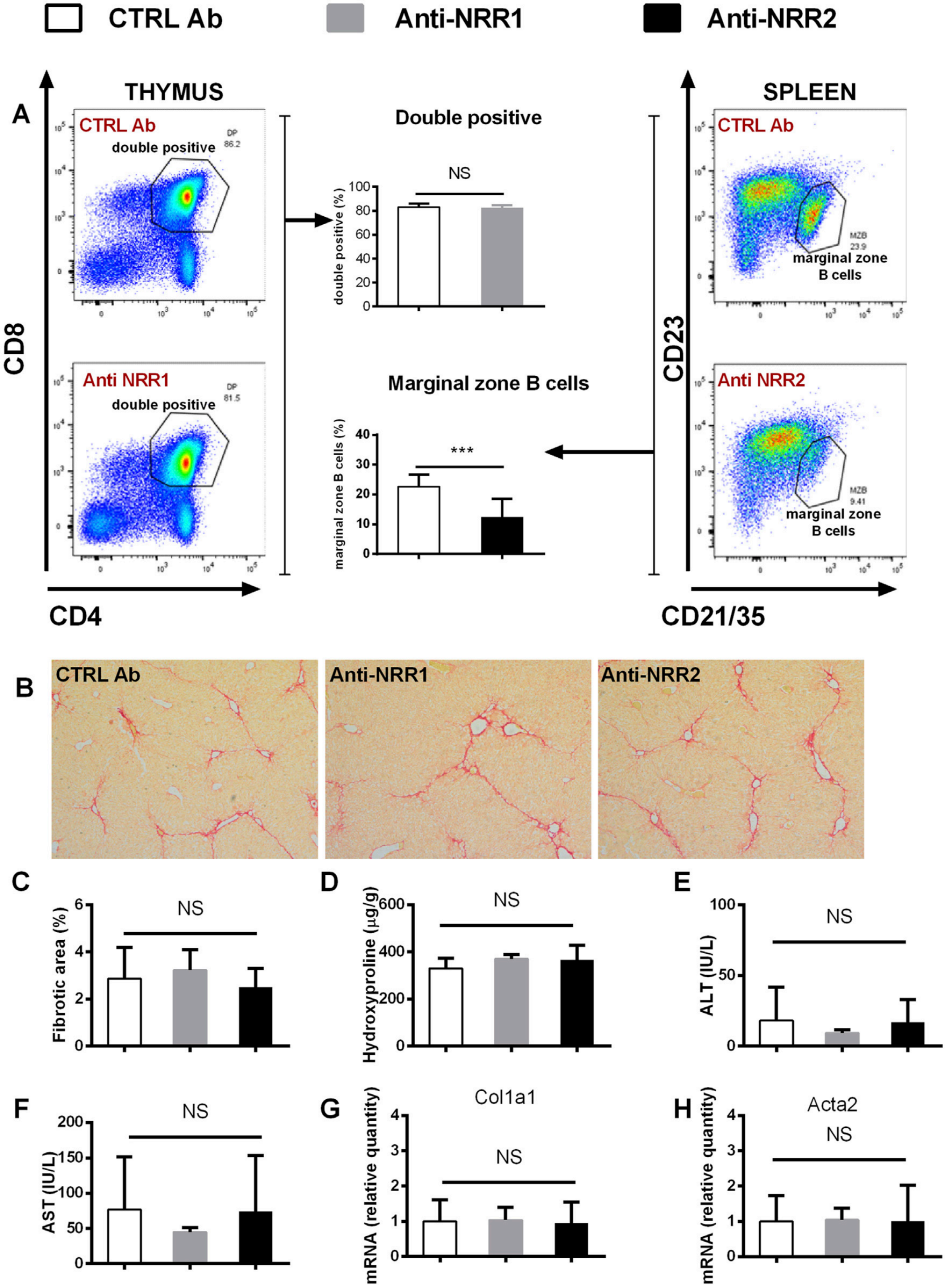
Antibodies against NOTCH1 and NOTCH2 receptors didn´t affect fibrosis development in a CCl4 model. Wild type mice (C57BL/6) were treated with CCl4 (2 x per week) and anti-NOTCH1 (Anti-NRR1. 1 mg/kg/2x per week) anti-NOTCH2 (Anti-NRR2, 2 mg/kg/2x per week) or control antibody (CTRL Ab, 2 mg/kg/2x per week) for 3 weeks. **(A)** Flow cytometry analysis of double positive (DP) thymocytes in thymus (left panel) and marginal zone B cells in spleen (right panel). **(B, C)** Sirius red stained area **D** hydroxyproline content in liver **(E)** ALT, and **(F)** AST aminotransferase activity in plasma **(G)** expression of genes *Col1a1* and **(H)** *Acta2* were analyzed (n = 6-8 per group). ANOVA test with Dunnett’s *post hoc* correction was used for comparison between the groups ***p< 0,001 NS, non-significant.

**FIGURE 7:**
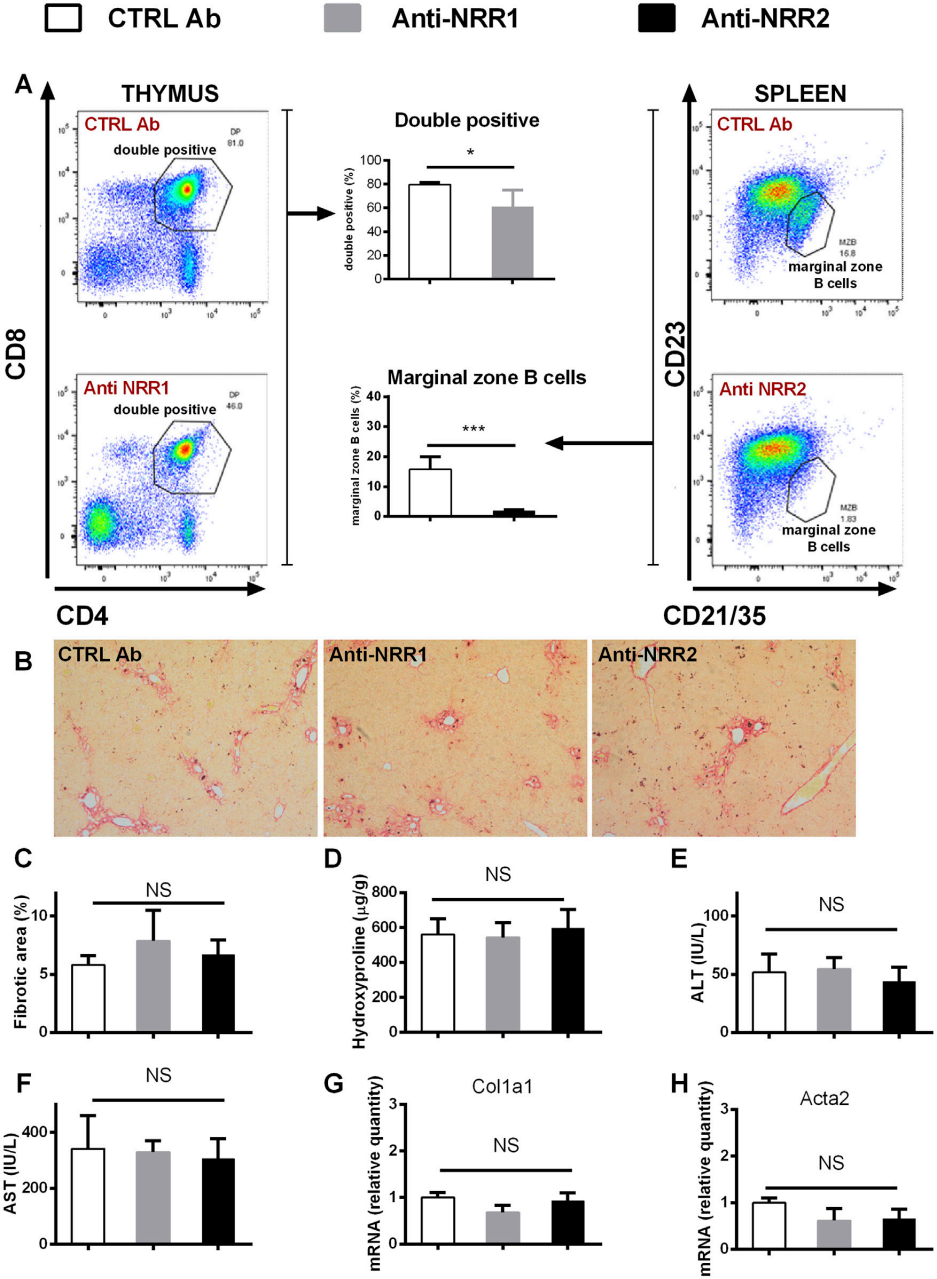
Antibodies against NOTCH1 and NOTCH2 receptors didn´t affect fibrosis development in a DDC model. Wild type mice (C57BL/6) were fed with DDC diet, and treated with anti-NOTCH1 (Anti-NRR1, 1 mg/kg/2x per week) anti-NOTCH2 (Anti-NRR2, 2 mg/kg/2x per week) or control antibody (CTRL Ab, 2 mg/kg/2x per week) for 3 weeks. **(A)** Flow cytometry analysis of double positive (DP) thymocytes in thymus (left panel) and marginal zone B cells in spleen (right panel). **(B, C)** Sirius red stained area **(D)** hydroxyproline content in liver **(E)** ALT, and **(F)** AST aminotransferase activity in plasma **(G)** expression of genes *Col1a1* and **(H)** *Acta2* were analyzed (n = 4 per group). ANOVA test with Dunnett’s *post hoc* correction was used for comparison between the groups *p< 0,05 ***p< 0,001 NS, non-significant.

In addition to genetic manipulation, we have investigated the effect of antibodies against NOTCH1 and NOTCH2 on the development of liver fibrosis. In the initial experiment antibodies (anti-NOTCH1, anti-NOTCH2 or stable mIgG2a (control antibody)) were all given at a dose of 2 mg/kg, which is well tolerated in animals without liver injury. However, the combination of anti-NOTCH1 treatment and liver fibrosis was associated with significant deterioration in animal health, so the animals were euthanized after 7 days due to ethical reasons (humane end-point criteria for the experiments were reached because of weight loss and impaired general condition). Therefore, in further experiments we decreased the dose of anti-NOTCH1 to 1 mg/kg which was well tolerated. The antibodies had effect on extrahepatic organs as evidenced by a decrease in the proportion of splenic marginal zone B cells in the anti-NOTCH2 treated groups (in both models Figures 6A, 7A) and a decrease in proportion of double positive thymocytes in a DDC model (Figure 7A), which was absent in the CCL4 model (Figure 6A). However, treatment with either anti-NOTCH1 or anti-NOTCH2 antibodies did not change the degree of liver fibrosis in either CCl4 (Figures 6B–H) or in DDC-model (Figures 7B–H).

## Discussion

In the present research, we used two models of liver injury to investigate how modulation of Notch-pathway regulates fibrogenesis. Although we confirmed that Notch-pathway is upregulated during the fibrogenesis, we found no difference between the Notch-modulated and control groups in various approaches. Our results are in contrast to the results of Yue et al. who showed that inducible inhibition of Notch in SMAα22 RBP-Jfl/fl positive myofibroblasts cells is sufficient to ameliorate liver fibrosis in the CCl4 model (Yue et al., 2021). The discrepancy might be the result of different promoter used to label HSC and application regimen, even though the expression of SMAα22 and αSMA appears to be overlapping in liver. Beside the genotype, a difference in the treatment regiments and tamoxifen application protocol might also be the reason for the contrasting results. We have previously shown that tamoxifen treatment prevents liver fibrosis in a cholestatic DDC model (Šisl et al., 2022), and similar effects were also reported in the CCl4 model (Hammad et al., 2018). Therefore, to avoid bias, in our experiments we used αSMACre+/RBP-Jfl/fl animals in which Notch signaling was inhibited by tamoxifen treatment and identically treated αSMACre-/RBP-Jfl/fl animals and didn’t find a difference in the degree of fibrosis between the groups. We conclude that Notch inhibition in αSMA positive cells is not sufficient to ameliorate liver fibrosis.

We further hypothesized that forced activation of Notch1 in αSMACre+/NICD1 mice will aggravate liver fibrosis. However, in the experiments with overactivation of Notch1 pathway we found similar degree of fibrosis in control animals and animals with Notch overactivation. Furthermore, we showed that if fibrotic stimulus is suspended the animals are able to restore normal liver architecture despite Notch1 overactivation. These results indicate that persistent activation of Notch1 pathway is not sufficient to sustain collagen secreting activity in activated HSC. Zhu et al. have shown that constitutional forced activation of Notch in hepatocytes exacerbates fibrosis in a model of nonalcoholic steatohepatitis, but the effect was not found in a methionine-choline deficient model (Zhu et al., 2018). To the best of our knowledge this is the first study that investigated the effect of inducible forced Notch activation in activated HSC. As we found no change in two common models of fibrosis it would be worthwhile to investigate other models of liver fibrosis in future research.

NOTCH1 and NOTCH2 receptors have been implicated as a promising target for the treatment of fibrosis of various organs (Hu and Phan, 2016; Huang et al., 2018; Xu et al., 2021), but the application is limited by the side effects. In our experiments, we had to reduce the dose of anti-NOTCH1 antibody because it negatively affected the animal health in fibrotic models. Applied doses of anti-NOTCH1 and anti-NOTCH2 antibodies were able to cause depletion of thymocytes and marginal zone B lymphocytes, but were not able to prevent development of fibrosis. Interestingly, the effect of anti-NOTCH1 antibodies on thymocytes was less pronounced in the CCl4 model than in the DDC model. As previously published research described effects of CCl4 on thymus (Chirculescu and Laky, 1978; Jirova et al., 1996), we can speculate that CCl4 treatment might affect sensibility of thymocytes, but the details of this interference remain to be investigated. Furthermore, the effects of inhibitory antibodies against NOTCH1 or NOTCH2 might be effective in other models of liver fibrosis.

Our study has several limitations. We used an inducible mouse model that allows modulation of Notch at a specific time point, which is important advantage to the constitutional modulation. However, the downside of this approach, is that recombination does not occur in all cells expressing the promoter, which might reduce the effect of modulation (Donocoff et al., 2020). We also tested inhibitory antibodies against NOTCH1 and NOTCH2 receptors, which were recently developed to specifically target NRR region of Notch1 or Notch2 and shown to induce more specific inhibition than γ-secretase inhibitors (Wu et al., 2010). Although, upregulation of Notch ligands was present in the investigated models of our study, the most upregulated receptor was NOTCH3 indicating that NOTCH3 inhibition could be interesting to investigate. Unfortunately, the inhibitory antibody for NOTCH3 is currently not commercially available. There is also a possibility that tamoxifen treatment, depletion of double positive thymocytes or marginal zone B cells interfered with mechanism of fibrogenesis.

In conclusion, our data demonstrate that modulation of Notch activity in injury activated HSC is not sufficient to change the outcome of liver fibrosis. The results obtained with inhibitory antibodies further demonstrate limitations of targeting NOTCH 1 and 2 receptors as a promising antifibrotic therapy. It is possible that potential beneficial effects are model-dependent, but the extrahepatic side-effects are also a limiting factor that should be considered in further investigations. Notch pathway remains a potential target for the treatment of liver fibrosis, but finding the appropriate application regimen continues to be a research challenge.

## Data Availability

The raw data supporting the conclusions of this article will be made available by the authors, without undue reservation.
