# Supplementary material for: Modulation of Notch signaling pathway in activated hepatic stellate cells does not ameliorate the outcome of liver fibrosis in carbon tetrachloride and DDC-feeding models
Source: Front Pharmacol. 2024 Oct 28;15:1440236. doi: 10.3389/fphar.2024.1440236 (PMC11551037; doi:10.3389/fphar.2024.1440236)
Supplement: Supplementary file 1 [file DataSheet1.docx]

**
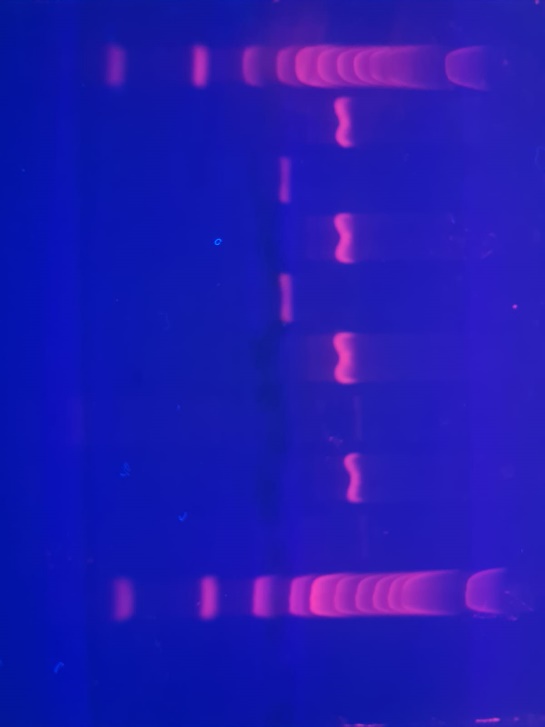
**

**Figure S1.** Complete photograph of the PCR represented in Figure 4A.


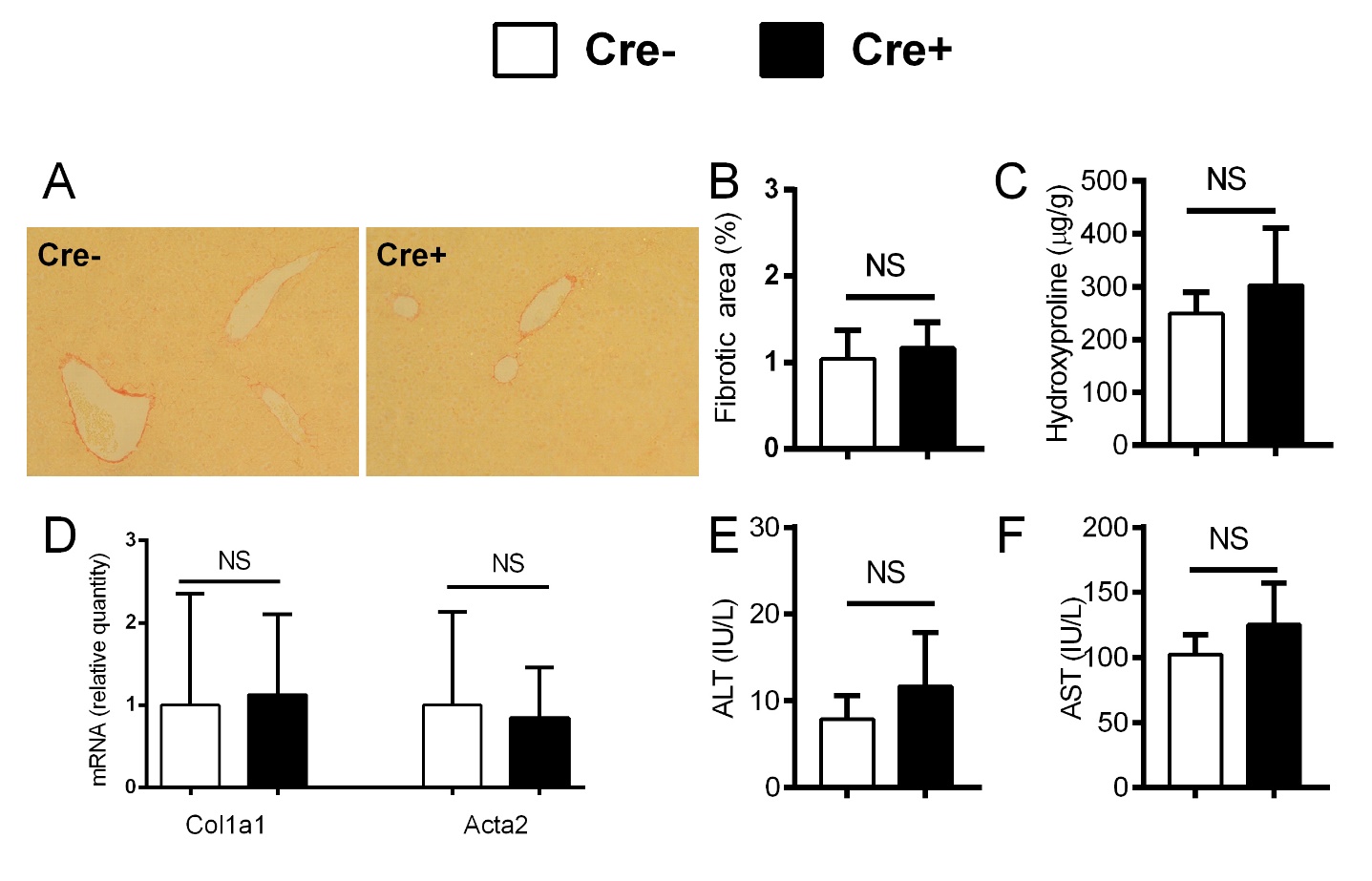


**Figure S2.** Forced activation of Notch1 in αSMA cells does not interfere with the recovery from fibrosis in DDC model A-F, Cre- and Cre+ aSMACreERT2/NICD mice were fed with DDC-diet and treated with TMX (3 x per week) for two weeks and then allowed to recover for four weeks, sirius red area (A, B), hydroxyproline content in liver (C), expression of genes Col1a1 and Acta2 (D) and ALT (E) and AST (F) aminotransferase activity in plasma were analyzed (n=6-8 per group). Data represent mean with Standard deviation, Student T test was used was used for comparison between the groups. NS, non-significant.
